# Metallic Burden of Deciduous Teeth and Childhood Behavioral Deficits

**DOI:** 10.3390/ijerph120606771

**Published:** 2015-06-15

**Authors:** Tony J.H. Chan, Carolina Gutierrez, Oladele A. Ogunseitan

**Affiliations:** 1 School of Social Ecology, University of California, Irvine, CA 92697, USA; E-Mail: tonyhp888@gmail.com; 2 School of Social Sciences, University of California, Irvine, CA 92697, USA; E-Mail: carolh2ogutierrez@gmail.com; 3 Department of Population Health and Disease Prevention, Program in Public Health, University of California, Irvine, CA 92697, USA

**Keywords:** ADHD, Behavior, Children, Pollution, Teeth, Metals

## Abstract

Attention Deficit/Hyperactivity Disorder (ADHD) affects 5%–8% of children in the U.S. (10% of males and 4% of females). The contributions of multiple metal exposures to the childhood behavioral deficits are unclear, although particular metals have been implicated through their neurotoxicity. The objective of this study was to test the hypothesis that the body burden of Mn is positively correlated with ADHD symptoms. We also investigated the putative roles of Ca, Fe, Pb, and Hg. We collected shed molars from 266 children (138 boys and 128 girls) who lost a tooth between 11 and 13 years of age. The molars were analyzed for metals using ICP-OES. The third grade teacher of each child completed the Teacher’s Disruptive Behavior Disorders Rating Scale (DBD) to produce a score for “Total Disruptive Behavior” and subscale scores for “Attention Deficit Hyperactivity Disorder”, Hyperactivity/Impulsivity, Inattention, and Oppositional/Defiant. The mean Mn, Fe, Pb and Ca concentrations found in teeth was 3.1 ± 2.9 µg/g, 11.4 ± 12.1 µg/g, 0.5 ± 0.7 µg/g, and 3.0 × 10^5^ ± 0.8 × 10^5^ µg/g, respectively. Hg was not detected. No significant association was found between Mn and behavioral deficits. Ca was significantly negatively associated, and Pb showed a significant positive association with Hyperactivity/Impulsivity, Inattention, and Oppositional/Defiant Disorders. These findings call into question the putative independent association of manganese exposure and behavioral deficits in children, when the balance of other metallic burden, particularly Ca and Pb burdens play significant roles.

## 1. Introduction

Attention-Deficit/Hyperactivity Disorder (ADHD) affects 5%–8% of children in the United States (10% of males and 4% of females) (CDC 2002). A definitive cause of ADHD has yet to be discovered, but some of the most promising research on the etiology of ADHD involves investigating the associations between the disorder and exposure to environmental risk factors. One of the potential risk factors is exposure to environmental manganese [1,2].

Manganese (Mn) is a ubiquitous and naturally occurring element found in certain rocks, making up about 0.1% of the earth’s crust [3]. Pure Mn does not occur naturally, but is a component of more than 100 minerals formed when Mn combines with other substances such as oxygen, sulfur, and chlorine. Mn compounds can change naturally or artificially from one compound to another, but the metal is generally stable in the environment [4,5,6]. Exposure to Mn can occur through inhalation or ingestion, but for most people, exposure to Mn is usually through diet because Mn is present in many foods, including tea, grains and cereals, and in drinking water [7]. Human physiology requires a certain amount of Mn, but the internal homeostasis is strictly regulated [8,9]. It is estimated that the average daily diet of an adult provides a range of 1–10 mg of Mn [10,11]. The National Research Council recommends a safe and adequate daily intake level of 5 mg/day for adults [12]. For children, the Food and Nutritional Board of the National Research Council considers daily intake levels of 0.3 to 1 mg/day for children under one years of age, 1 to 2 mg/day for children under 10, and 2 to 5 mg/day for children 10 and older to be safe and adequate [4].

In animals, exposure to excess Mn has been associated with neurochemical deficits [13,14,15], neuroanatomical deficits [16,17], neonatal deficits, and executive function deficits [15]. The first reported cases of Mn toxicity in humans occurred among Scottish workers exposed to dusts of grinding Mn ores who developed “a bizarre disorder with both neurological and behavioral components” [18]. These behavioral conditions resulting from chronic exposure to Mn became diagnosed as “manganism” or Mn poisoning. In 1977, the first study was published linking sub-clinical Mn toxicity to children’s behavior deficits [19]. A study by Hafeman *et al.* [20] found that infants exposed to water with manganese levels greater than the 2003 World Health Organization standard of 0.4 mg/L had higher mortality risk during their first year of life compared to unexposed infants.

The underlying mechanism(s) through which excess Mn is linked to behavioral disorders is not fully understood. Some scientists speculate that the abnormal behaviors are due to the toxic effects of manganese on the dopaminergic system [21]. The fact that Mn accumulates in dopaminergic areas has prompted studies to determine whether Mn neurotoxicity affects dopaminergic neurotransmission [22]. In vitro studies show that Mn toxicity can decrease intracellular dopamine levels by autoxidation [13]. The resulting Parkinson-like condition demonstrates that “Mn is specifically toxic to the brain’s dopamine systems” [22]. A similar study implicated Mn in causing the decay of dopamine by catalyzing the oxidation of the dopamine ligand [23]. This is similar to the neurotoxic effect of impaired dopaminergic functioning seen in children with ADHD. New-born animals that receive excess doses of Mn during their first few weeks of life show abnormalities in the dopaminergic nigrostratial system. These animal studies have shown that Mn exposure can lead to neurotoxic damage or dopamine deficits in the dopaminergic systems within the brain [24,25].

There are several methods for measuring Mn concentrations in humans. Mn in the blood is the most widely accepted method of measurement. The main limitation of using blood is that it only provides a reliable indication of recent exposure to Mn. The estimated half-life of Mn in the body is 37 days [26]. For records of exposure over longer periods of time, hair has been utilized, because hair protein absorbs excreted Mn from diet [2,27]. But similar to blood, the exposure assessment from hair is generally short, with measurements accounting for an exposure history of only a few months on average [27]. Neutron activation analysis of the liver has also been conducted to determine recent Mn exposure [28]. In contrast, analysis of the deciduous tooth enamel allows measurements of Mn exposure, going as far back as the 20th week gestation and as recent as 63rd gestational week [29] because calcified tissues incorporate heavy metals to which they are exposed during development [30]. This is important as the time frame coincides with neuronal development occurring within the brain [31]. Complete tooth formation occurs during the early postnatal stages of a child’s development, but tertiary or reactive/reparative dentin is continually produced when needed, such as replacing existing dentin destroyed by caries. In addition, subsurface enamel loss from such events as caries can be remineralized from Ca and phosphate ion in saliva as long as there is no breakdown of the surface layer. During the reparative and remineralization process, Mn and other metals are deposited in teeth just as it would during tooth development. During tooth formation and mineralization, Ca absorbed can be partially substituted by heavy metals [32] and the relative stable nature of dental tissue allows a large extent of these metals deposited in the teeth during mineralization to be retained [33].

Some published studies have shown significant correlations between elevated Mn hair levels and increased expression of behavioral deficits in children [2,34,35]. There have also been studies published using human teeth as a potential biomarker of environmental exposure to heavy metals [1,36,37], but few have then concurrently compared the measured Mn exposure to behavioral measurements of the subjects. Findings from a study using 27 teeth from 27 children as a biomarker for Mn exposure suggested that prenatal accretion of Mn in tooth enamel is significantly associated with more frequent hyperactive, impulsive, inattentive, aggressive, defiant, destructive and disobedient behavior [29]. This study expands upon these previous experiments in investigating the role of Mn in the development of ADHD and other behavioral deficits by specifically testing the hypothesis that high body burden of manganese is positively correlated with ratings of ADHD.

In addition to Mn concentrations, the levels of iron (Fe), calcium (Ca) and lead (Pb) and mercury (Hg) within the teeth were investigated in order to assess their putative roles independently or in interactive ways. Fe and Ca are of particular interest because studies indicate that Mn absorption in the human body can be affected by deficiencies in Fe [38,39] and Ca [40]. Fe deficiency is considered the most widespread nutritional deficiency among children [41,42] and Mn is initially absorbed via the same biological pathway as Fe (*i.e*., the intestine does not differentiate between Mn and Fe) [43,44,45]. Evidence from animal studies indicates that Mn displays an inverse relation with dietary Fe concentrations [4]. Therefore, dietary Fe deficiency leads to increased Mn absorption and toxicity whereas high levels of Fe lead to decreased Mn absorption and toxicity [46,47,48,49]. Conversely, high levels of dietary Mn lead to lower Fe absorption [43,49]. Fe deficiency has also been correlated with child behavioral abnormalities [50]. Short-term causes of the relation between Fe and Mn are believed to be the result of kinetic competition between the two metals for available binding sites on intestinal transport enzymes [51] while long-term causes are believed to be the result of adaptive changes in intestinal transport capacity [18]. Competition between Fe and Mn has been reported to extend to the blood-brain barrier, indicating that high levels of either metal will affect the brain distribution of the other metal [52].

Calcium deficiency has been suggested in animal studies to cause Mn neurotoxicity by increasing dietary Mn absorption and Mn levels in the brain e.g. [40]. A similar study found that rats fed a low Ca diet showed significantly higher Mn levels in the frontal cortex and suggested that these types of unbalanced mineral diets and metal-metal interactions may lead to CNS degeneration [53]. Pb was also included in the study because Pb toxicity in biological systems has been extensively documented [54] and low-level exposure has also been associated with cognitive and behavioral deficits in children [55]. Determining the concentration levels of these additional elements linked to behavioral disorders will aid in establishing whether these elements are potential causes in the behavioral deficits observed in this study population.

## 2. Materials and Methods

Shed molars were collected from 266 children in the NICHD Study of Early Child Care and Youth Development (SECCYD) who lost a tooth between 11 and 13 years of age. The parents voluntarily submitted the teeth, which were subsequently stored in snap-lock plastic containers. The families that donated teeth were not significantly different from the other 714 families in the SECCYD sample in terms of family income, ethnicity, or child behavioral outcomes. However, the parents who donated their child’s tooth had significantly higher education levels (14.7 years *vs.* 14.1 years, *p* < 0.05).

### 2.1. Behavioral Measures

Third grade teachers completed the Disruptive Behavior Disorders (DBD) Rating Scale. Twenty-six behavior items were scored on a 4-point scale composed of 0 = not at all, 1 = just a little, 2 = pretty much, 3 = very much. The DBD scale was derived from the Diagnostic and Statistical Manual of Mental Disorders (DSM-IV) and produces a Total Disruptive Behavior Score as well as subscale scores for ADHD, Inattention, Hyperactivity/Impulsivity, and Oppositional Defiance [56,57]. The Disruptive Behavior Disorder Total Score is computed as the sum of the items 1 to 26. The possible scores range from 0 to 78, with higher scores indicating more hyperactive-impulsive, inattentive, and oppositional defiant behaviors. The Oppositional Defiant Score is computed as the sum of eight items producing a score ranging from 0 to 24. Hyperactive-Impulsive and Inattentive Scores are computed as the sum of nine separate items for each behavior type. The possible scores for both forms of behavior are 0 to 27. The ADHD score is computed as the sum of the 18 hyperactive and inattentive items. The possible scores range from 0 to 54, with higher scores indicating more attention deficit-hyperactive behaviors. The reliabilities of these scales (Cronbach’s alphas) are 0.96 for the 26-item Total Disruptive Behavior Disorder Score, 0.95 for the 18-item Attention Deficit/Hyperactivity Disorder Score, 0.93 for the 9-item Hyperactive-Impulsive Score, 0.95 for the Inattentive Score, and 0.93 for Oppositional Defiant Score. Pelham *et al.* [68] reported internal consistency of 0.96, 0.95, and 0.95 for Oppositional Defiance, Inattention, and Hyperactivity-Impulsivity respectively.

### 2.2. Preparation and Analysis of Teeth

Two hundred and sixty six molar teeth were longitudinally sectioned with a diamond blade on an Isomet low speed saw (Buehler, Lake Bluff, IL, USA). One half of the molar tooth was utilized for metal concentration analysis. Tooth samples were washed thoroughly with distilled deionized water. The samples were first air-dried and then later dried at 85 ºC to a constant mass before being pulverized using a glass pestle and mortar. After pulverization, each sample was further dried at 85 ºC for 240 min and weighed prior to digestion [37].

Manganese (Mn), iron (Fe), calcium (Ca), mercury (Hg) and lead (Pb) concentrations were determined through Inductively Coupled Plasma-Optical Emission Spectroscopy (ICP-OES). EPA 6010 Method was used to determine Mn, Fe, Ca, and Pb concentrations; EPA 7471 Method was used for Hg. Sensitivity limits of the ICP-OES are 0.01 mg/L for Mn, Fe, Ca, Pb and 0.0002 mg/L for Hg. The weights for the sample teeth ranged from 120 to 580 mg per tooth sample with an average of 280 mg. A sample weighing 250 mg of dried powdered tooth was digested with 1.5 mL of concentrated nitric acid in a Teflon vessel at 120 ºC for 180 min before being diluted with distilled water to 5 mL. The volume of concentrated nitric acid and distilled water were adjusted accordingly for samples weighing less or more than 250 mg. Instrument-calibrated element standards were used as controls for experimental sample measurements. The measured metal concentrations were not adjusted for teeth dry weight or for loss of organic matter during the digestion procedure.

### 2.3. Statistical Analyses Linear Associations

SPSS version 10.0 (SPSS, Chicago, IL, USA) was used for the statistical analyses. Three sets of linear regression models were fit to determine whether a significant association exists between the concentration of each metal and the behavioral deficit assessments (*p* = 0.05 level of significance). The first set of analyses evaluated the bivariate associations. The second set of analyses controlled for the child’s race, gender, paternal education, maternal education, marital status of parents, and family social economic status. The third set of regression models determined whether there was an association between each metal and the behavioral measurements controlling for the other four elements.

### 2.4. Dichotomous Comparisons

Unpaired t tests were performed comparing the 50 subjects with the highest DBD, ADHD, Inattention, Hyperactivity/Impulsivity, and Oppositional Defiant scores to 50 subjects with DBD, ADHD, Inattention, Hyperactivity/Impulsivity, and Oppositional Defiant scores of zero. Unpaired t tests were also performed comparing subjects who meet the criteria for DSM-IV diagnoses of DBD, ADHD, Inattention, Hyperactivity/Impulsivity, and Oppositional Defiance to those subjects showing no symptoms of these disorders. Children who met the criteria for DSM-IV diagnoses scored two standard deviations above the normative mean obtained by the 980 subjects in the national sample participating in the NICHD Study of Early Child Care and Youth Development [57].

## 3. Results

The ratio of males to females in the study was 138 to 128 respectively. Of the participants, 234 identified as White, 19 as African American, 4 as Asian, and 8 as other. Eighty-four percent or 224 of the children came from families in which the parents were married and living together. The income of the families ranged from $2000 to $230,000 with an average of $56,988 and a median of $47,500. The number of teeth collected from each study site was as follows: Little Rock, AR = 26, Irvine, CA = 19, Lawrence, KS = 11, Boston, MA = 35, Pittsburgh, PA = 24, Philadelphia, PA = 26, Charlottesville, VA = 21, Seattle, WA= 40, Morganton, NC = 23 and Madison, WI = 41. These characteristics are summarized in Table 1.

**Table 1:** Descriptive characteristics of the 266 study participants.

Characteristic	Values		
Site Location and Number of Subjects	Charlottesville, VA = 21		
	Irvine, Ca = 19		
	Lawrence, KS = 11		
	Little Rock, AR = 26		
	Madison, WI = 41		
	Morganton, NC = 23		
	Philadelphia, PA = 26		
	Pittsburgh, PA = 24		
	Seattle, WA = 40		
	Boston, MA = 35		
Ethnicity	African American = 19		
	Asian = 4		
	Other = 8		
	White = 234		
Marital Status	Married = 224		
	Single = 42		
Gender	Male = 138		
	Female = 128		
	*Range*	*Average*	*Median*
Mother’s Education (Years)	7–21	14.9	14
Father’s Education (Years)	9–21	15.2	14
Income (US$)	2000–230,000	56,000	47,000

Results for behavioral measures and metal analysis are shown in Table 2. The children in this study had average behavioral scores of 11.60 ± 13.77 for DBD, 9.44 ± 11.01 for ADHD, 4.34 ± 5.82 for Hyperactivity/Impulsivity, 5.11 ± 6.22 for Inattention, and 2.16 ± 3.75 for Oppositional Defiance. The DBD total score for the children must be larger than their respective subscale scores, including ADHD. The mean Mn, Fe, Pb and Ca concentrations found in teeth was 3.1 ± 2.9 µg/g, 11.4 ± 12.1 µg/g, 0.5 ± 0.7 µg/g, and 3.0 × 10^5^ ± 0.8 × 10^5^ µg/g, respectively. Mercury was not detected in any of the teeth.

**Table 2:** Average behavioral measure scores and metal concentrations.

Statistical Parameter	Behavioral Measures					Metal Concentrations (µg/g)				
	Hyperactive				Oppositional	Ca	Fe	Pb	Mn	Hg
	DBD	ADHD	Impulsive	Inattention	Defiant					
N	266	266	266	266	265	265	261	266	262	266
Mean	11.60	9.44	4.34	5.11	2.16	3.18 × 10^5^	11.34	0.46	3.06	-
SD	13.77	11.01	5.82	6.22	3.75	0.84 × 10^5^	12.06	0.73	2.85	-

N—Sample size; SD—Standard Deviation.

### 3.1. Bivariate Associations 

Linear regression models indicated no significant association between the behavioral deficit scores and the concentrations of metals in teeth, with the exception of Ca (Table 3). Low levels of Ca were related to higher behavioral deficit scores: DBD (Beta = −0.25, *p* < 0.01), ADHD (Beta = −0.25, *p* < 0.01), Hyperactivity/Impulsivity (Beta = −0.25, *p* < 0.01), Inattention (Beta = −0.21, *p* < 0.01), Oppositional Defiance (Beta = −0.20, *p* < 0.01). Effect sizes using Cohen’s guidelines for a small (r^2^ = 0.01), medium (r^2^ = 0.06), large (r^2^ = 0.14) were of medium size for DBD, ADHD, and Hyperactivity/Impulsivity and a small to medium size effect for Inattention and Oppositional Defiance. Results for Fe approached significance for three of the behavioral scores, showing an inverse association with DBD (B = −0.10, *p* = 0.093), ADHD (B = −0.10, *p* = 0.091), and Hyperactivity/Impulsivity (B = −0.11, *p* = 0.07).

**Table 3:** Bivariate associations between the behavioral scores and metal concentrations.

Metal	Statistical Parameters	Hyperactive				Oppositional
		DBD	ADHD	Impulsive	Inattention	Defiance
Mn	Beta	0.06	−0.06	−0.08	−0.02	−0.07
	R^2^	0.004	0.003	0.007	0.001	0.004
	N	262	262	262	262	261
Ca	Beta	−0.25 ***	−0.25 ***	−0.25 ***	−0.21 ***	−0.20 **
	R^2^	0.004	0.003	0.007	0.001	0.004
	N	265	265	265	265	264
Fe	Beta	−0.10	−0.10	−0.11	−0.08	−0.08
	R^2^	0.011	0.011	0.013	0.007	0.006
	261	261	261	261	260	261
Pb	Beta	−0.05	−0.03	−0.06	0.00	−0.09
	R^2^	0.002	0.001	0.003	0.000	0.008
	N	266	266	266	266	265

*p* < 0.05; ** *p* < 0.01; *** *p* < 0.001.

### 3.2. Adjusted Effects

Linear regression models controlling for race, sex, paternal education, maternal education, marital status of parents, and family social economic status produced similar results. Ca was the only metal showing a significant association with the behavioral scores, with the exception of Inattention (see Table 4). The beta coefficients for Ca were slightly smaller in magnitude: DBD (Beta = −0.15, *p* = 0.014), ADHD (Beta = −0.15, *p* = 0.017), Hyperactivity/Impulsivity (Beta = −0.17, *p* < 0.01), Oppositional Defiance (Beta = −0.12, *p* = 0.05), and Inattention (Beta = −0.10, *p* = 0.10). Results for Fe no longer approached significance when controlling for the covariates. Similarly, linear regression models showed Ca being the only metal to show a significant association with the behavioral scores when controlling for the other three metals (see Table 5): DBD (Beta = −0.238, *p* < 0.001), ADHD (Beta = −0.234, *p* < 0.001), Hyperactivity/Impulsivity (Beta = −0.236, *p* < 0.001), Oppositional Defiance (Beta = −0.195, *p* = 0.003), and Inattention (Beta = −0.189, *p* = 0.004).

**Table 4:** Association between behavior scores and metal concentrations controlling for covariates ^a^.

Metal	Statistical Parameters	Hyperactive				Oppositional
		DBD	ADHD	Impulsive	Inattention	Defiance
Mn	Beta	−0.011	−0.007	−0.039	0.025	−0.020
	N	231	231	231	231	231
Ca	Beta	−0.152 *	−0.15 *	−0.171 **	−0.105	−0.119 *
	N	233	233	233	233	233
Fe	Beta	−0.072	−0.078	−0.092	−0.051	−0.04
	N	230	230	230	230	230
Pb	Beta	−0.027	−0.006	−0.023	0.011	−0.080
	N	234	234	234	234	234

^a^ controlling for race, sex, paternal education, maternal education, marital status of parents, and family social; economic status; * *p* < 0.05; ** *p* < 0.01.

**Table 5:** Association between behavior scores and individual metal concentrations controlling for the other four metals.

Metal	Statistical Parameters	Hyperactive				Oppositional
		DBD	ADHD	Impulsive	Inattention	Defiance
Mn	Beta	0.051	0.056	0.035	0.067	0.026
	N	260	260	260	260	260
Ca	Beta	−0.238 ***	−0.234 ***	−0.236 ***	−0.195 **	−0.189 **
	N	260	260	260	260	260
Fe	Beta	−0.078	−0.081	−0.080	−0.070	−0.049
	N	260	260	260	260	260
Pb	Beta	−0.009	0.008	−0.022	0.035	−0.055
	N	260	260	260	260	260

*p* < 0.01; *** *p* < 0.001.

### 3.3. Comparison of Metal Levels for Low and High Behavioral Deficit Groups 

**Figure 1:**
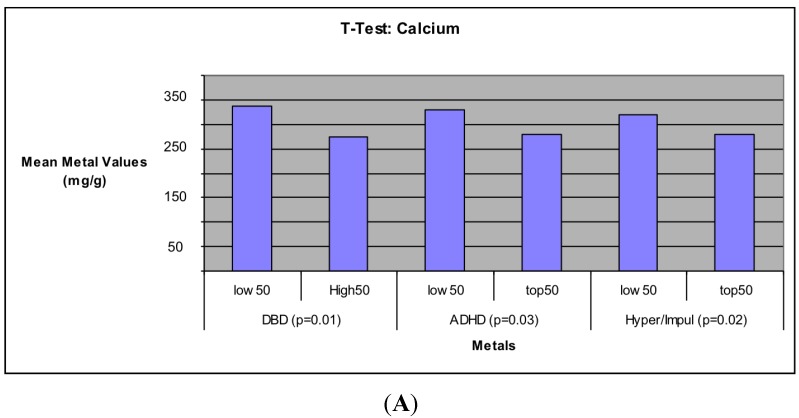

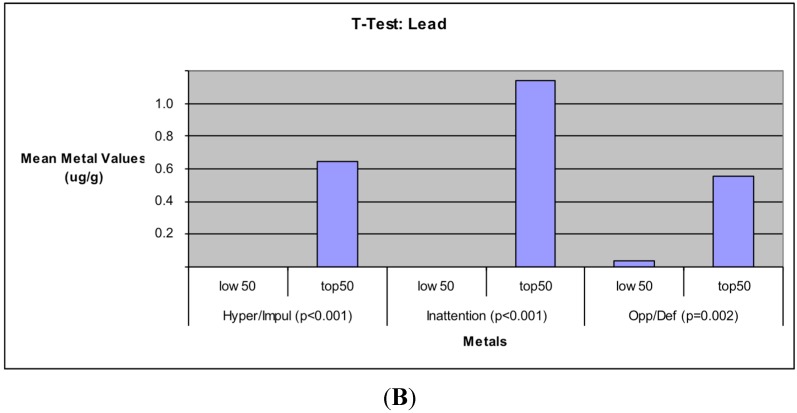
Significant mean metal concentrations of Ca (**A**) and Pb (**B**) for the 50 subjects with the highest behavioral scores and the 50 subjects with the lowest behavioral scores.

Additional analyses were conducted to compare concentrations of metals for the 50 subjects with the highest and the lowest behavioral scores (Table 6). Results of unpaired t-tests indicated no significant differences in Mn and Fe. Ca was significantly higher in the group with the lowest when compared to the group with the highest behavior scores for three of the behavior measurements, DBD, ADHD, and Hyperactivity/Impulsivity (see Figure 1A). Pb was significantly higher in the group with the highest scores for Inattention, Hyperactivity/Impulsivity, and Oppositional Defiance (see Figure 1B). Using Cohen’s guidelines for a small (r^2^ = 0.01), medium (r^2^ = 0.06), large (r^2^ = 0.14) effect sizes were moderate. Unpaired t-tests conducted to compare subjects who met the criteria for DSM-IV diagnosis of DBD, ADHD, Inattention, Oppositional Defiance, and Hyperactivity/Impulsivity to subjects showing no measured symptoms of the respective disorders did not attain significance.

**Table 6:** Mean metal concentrations (µg/g) for the 50 subjects with the highest behavioral scores and the 50 subjects with the lowest behavioral scores (SD—Standard Deviation).

**Behavioral Score (Top 50)**	**Metallic Burden and Standard Deviation**							
	**Mn**	**SD**	**Fe**	**SD**	**Pb**	**SD**	**Ca**	**SD**
DBD	2.82	3.75	8.86	6.71	0.40	0.64	2.75 × 10^5^	1.25 × 10^5^
ADHD	2.77	3.69	8.58	6.92	0.45	0.84	2.79 × 10^5^	1.26 × 10^5^
Hyperactive/Impulsive	2.41	3.00	9.01	8.81	0.32	0.59	2.78 × 10^5^	0.76 × 10^5^
Inattention	3.02	3.73	0.57	6.40	0.57	0.93	2.93 × 10^5^	1.15 × 10^5^
Oppositional/Defiant	2.56	2.65	9.05	7.95	0.28	0.54	2.89 × 10^5^	1.01 × 10^5^
**Behavioral Score (Bottom 50)**	**Metallic Burden and Standard Deviation**							
	**Mn**	**SD**	**Fe**	**SD**	**Pb**	**SD**	**Ca**	**SD**
DBD	3.09	2.73	12.45	18.23	0.59	0.82	3.37 × 10^5^	1.09 × 10^5^
ADHD	2.91	2.40	12.22	18.30	0.49	0.72	3.30 × 10^5^	1.05 × 10^5^
Hyperactive/Impulsive	3.19	2.57	13.11	16.78	0.00	0.00	3.18 × 10^5^	0.85 × 10^4^
Inattention	3.11	2.75	10.77	15.55	0.00	0.00	3.19 × 10^5^	0.79 × 10^4^
Oppositional/Defiant	3.36	3.14	13.54	16.89	0.21	0.14	3.15 × 10^5^	0.85 × 10^4^

## 4. Discussion

The results of this study indicate that there is no significant association between Mn levels found in deciduous teeth and measurements of behavioral deficit in children. The results did not achieve statistical significance even after adjustment for race, sex, paternal education, maternal education, marital status of parents, and family social economic status. The elements Ca and Fe, previously shown to influence Mn absorption in the human body were added to regression models to help determine if the association between Mn and the behavior measurements may have been modified by these two environmental risk factors [38,40,58]. These findings contradict previous studies that indicate a significant association between elevated Mn concentrations and behavioral deficits in children [2,59].

There was no significant association found between Fe levels in the teeth and the behavioral measurements. These results also contradict previous studies that suggest low Fe levels contribute to ADHD symptoms [60,61]; instead supporting opposing studies that indicate Fe deficiency does not contribute to ADHD [62]. No statistical analysis was performed to test the relationship between Hg and the behavioral measurements as Hg levels in the teeth were below the detection limit of the instrument.

The results showed significant negative associations between Ca concentration levels and all five behavioral measurements, indicating that higher levels of Ca was associated with decreased occurrences of behavioral problems measured in the children. These relations remained significant after adjustment for the other metals as well as race, sex, paternal education, maternal education, marital status of parents, and family social economic status. Unpaired t-test tests comparing the 50 subjects with the highest behavioral scores to the 50 with the lowest also showed this same significant negative relation, but only for three of the behavior measures (DBD, ADHD, and Hyperactivity/Impulsivity). Further study is needed to determine why Inattention and Oppositional Defiance were not significant. These associations further support the importance of Ca in human physiology. The importance of Ca in promoting strong bones has been well documented [63,64,65,66], but this and other studies have also demonstrated the potential importance of Ca in regards to child behavior [67,68]. Studies have indicated that deficiencies in calcium and other biochemicals is associated with higher incidents of childhood behavioral deficits [68] while the activity of calcium channels has been suggested in helping reduce the negative symptoms of schizophrenia [67]. Furthermore, correlation models from this study show Ca levels positively correlated with higher levels of the other three metals (Table 7). This would indicate that children with higher Ca levels are probably more likely to be receiving a proper diet of other essential nutrients. This proper diet can help promote normal development in the children, thus, potentially explaining the lower levels of behavioral deficits observed in the children of this study.

**Table 7:** Bivariate correlations between Ca and other metals.

Metal	Statistical Parameter	Metal		
		Fe	Pb	Mn
Ca	Partial Correlation	0.190 **	0.191 **	0.346 ***
	Sample Size (N)	261	265	262

** *p* < 0.01; *** *p* < 0.001.

The additional findings in this study that elevated Pb levels is significantly associated with increased incidents of Hyperactivity/Impulsivity, Inattention, and Oppositional Defiance in children also support the well documented toxic effects of Pb on the human body [54] and its association with cognitive and behavioral deficits in children [55].

### 4.1. Limitations

There are several important limitations to this project. First, the participants were not randomly selected but were instead self-selected and it is possible that the parents who felt their children had no observable behavioral problem were more willing to send in the child’s tooth for analysis. In addition, the parents who donated their child’s tooth had significantly higher education levels (14.7 years *vs.* 14.1 years, *p* < 0.05) [29]. The more educated parents may have better understood the importance of submitting the teeth for analysis and were more dedicated to sending in samples. Second, there are presently no set low, medium or high concentration levels for these environmental risk factors within teeth. All concentrations measured can only be used relative to other samples in the study when analyzing teeth. Third, the tooth does not present an absolute log of the environmental exposure during the children’s lives. The tooth is biased toward the 20th week gestation to the 63rd gestational weeks of a child’s development [29]. Any time after this period is dependent on whether there was any tertiary repair of dentin or re-mineralization of the subsurface enamel. A child may display behavioral problems caused by acute or chronic exposure to environment toxins after the 63rd gestational week that may not be detected in the tooth. Fourth, although we took extensive methodological care to avoid cross-contamination across samples, and to avoid interference from tissue not associated with teeth, we are aware that other potentially aggressive methods such as hydrogen peroxide soaking are sometime used. We also note the large standard deviations in the metal measurements, and the fact that we did not correct for metal concentrations with respect to changes associated with tooth preparation processes, including drying, grinding, and acid digestion, and we relied on calibrated instrument standards as controls. We prioritized methodological standardization based on similar published research from our laboratory, and the results of our chemical analysis procedures are consistent with other published studies using teeth as a biomarker of exposure [1,37].

### 4.2. Implications 

Despite the limitations, the results of this study are valuable additions to the growing research in investigating the relationships between environmental toxicants and behavioral deficits, and the use of biomarkers to measure these associations. This study represents one of the largest sample sizes to date for this type of experiment, giving it greater statistical power. Although no significant associations were found between manganese and the behavioral deficit scores, the significant findings on Pb and especially Ca are further evidence of the important roles these two elements play in child development. The results of this and other studies have not only acknowledged the potential harms of Pb toxicity [54] but has also advanced the role of calcium in human physiology beyond bone growth and development and into the realm of cognitive function [67,68].

The tooth does not represent a perfect biomarker, but its proven effectiveness at accumulating and storing heavy metals [37] provides an effective method of measuring environmental exposure during the early stages of a child’s development. Recent studies support the view that Manganese levels in tooth dentine show promise as a biomarker of perinatal exposure [69,70] but cannot make such a claim based on the present study because we did not conduct a comparative assessment of biomarkers. Biomarkers such as hair and blood are reputably effective at measuring recent exposure. Future research, if possible, should consider incorporating the use of both the subjects tooth and hair/blood as biomarkers. The combined usage of teeth to measure early exposure and hair/blood to measure recent exposure provides the most accurate and comprehensive log of a subject’s exposure history to environmental contaminates.

## 5. Conclusions

Unlike lead and mercury that are unequivocally toxic at all levels of exposure, manganese in diet is essential for normal human development and maintenance of health. Healthy individuals with normal physiological functions can regulate excessive levels of required metals such as calcium, iron, and manganese. However, excessive levels of manganese and routes of exposure such as inhalation are known to elicit adverse health outcomes. Previous research suggested that manganese accumulation in teeth is linked to measurable behavioral deficits in children. Through this research, based on a larger sample size than previously possible, we found no significant relationship between dental manganese and behavioral deficits, and we reconfirmed the adverse impacts of lead exposure. The detected protective effect of calcium levels warrants further investigation, and the results on manganese has implications for attempts to regulate the acceptable levels of manganese in water supply and diet components.
